# COVID-19 vaccination and clinical outcomes of immune checkpoint inhibitors therapy in cancer patients: a meta-analysis of real-world studies

**DOI:** 10.3389/fimmu.2026.1807267

**Published:** 2026-04-16

**Authors:** Hongyan Xi, Meichao Li, Jinhai Shen, Yuxuan Lin, Beibei Wang, Qingyun Yu, Hongmei Wang, Yuxin Zhao

**Affiliations:** 1 Department of Obstetrics and Gynecology, Yancheng TCM Hospital Affiliated to Nanjing University of Chinese Medicine, Yancheng, Jiangsu, China; 2 Department of Obstetrics and Gynecology, Yancheng TCM Hospital, Yancheng, Jiangsu, China; 3 Department of Integrated Traditional Chinese and Western Medicine, Liuzhou People’s Hospital, Liuzhou, Guangxi, China; 4 State Key Laboratory of Natural Medicines, China Pharmaceutical University, Nanjing, Jiangsu, China; 5 Center for New Drug Safety Evaluation and Research, China Pharmaceutical University, Nanjing, Jiangsu, China; 6 School of Basic Medicine and Clinical Pharmacy, China Pharmaceutical University, Nanjing, Jiangsu, China; 7 Department of Pharmacy, Guangxi Hospital Division of The First Affiliated Hospital, Sun Yat-sen University, Nanning, Guangxi, China

**Keywords:** cancer, COVID-19 vaccination, immune checkpoint inhibitors, meta-analysis, real-world evidence

## Abstract

**Background:**

Immune checkpoint inhibitors (ICIs) have revolutionized cancer therapy, yet treatment responses remain heterogeneous. With the widespread implementation of coronavirus disease 2019 (COVID-19) vaccination, uncertainty persists regarding its impact on antitumor efficacy in patients receiving ICIs. While vaccine safety has been extensively studied, the association between COVID-19 vaccination and ICI therapeutic outcomes has not been systematically evaluated.

**Methods:**

We conducted a systematic review and meta-analysis of observational studies examining the association between COVID-19 vaccination and oncologic outcomes in patients treated with ICIs. PubMed, Embase, and Scopus were searched from 2020 to December 31, 2025. Primary outcomes were overall survival (OS) and progression-free survival (PFS); secondary outcomes included objective response rate (ORR) and disease control rate (DCR). Hazard ratios (HRs) and odds ratios (ORs) with 95% confidence intervals (CIs) were pooled using random-effects models.

**Results:**

Ten observational studies comprising 4,929 patients receiving ICIs were included. COVID-19 vaccination was associated with significantly improved PFS (pooled HR = 0.66, 95% CI 0.48–0.90) and OS (pooled HR = 0.51, 95% CI 0.39–0.66) compared with no vaccination. Vaccinated patients showed numerically higher ORR (pooled OR = 1.74, 95% CI 0.89–3.41) and DCR (pooled OR = 1.74, 95% CI 0.83–3.46), although these differences were not statistically significant. Subgroup analyses by vaccine platform and cancer type yielded consistent associations.

**Conclusions:**

COVID-19 vaccination is associated with improved survival outcomes in patients receiving ICIs. Although the observational nature of available data warrants cautious interpretation, the consistency of findings and their biological plausibility support the clinical compatibility of vaccination with ICI therapy, but causal or synergistic effects cannot be established from these data. These results reinforce current vaccination recommendations and highlight the need for prospective studies to further elucidate underlying mechanisms and optimize integration with cancer immunotherapy.

**Systematic review registration:**

https://www.crd.york.ac.uk/prospero/, identifier CRD420261277938.

## Introduction

Immune checkpoint inhibitors (ICIs), particularly agents targeting the programmed cell death 1 (PD-1) and programmed cell death ligand 1 (PD-L1) pathways, have fundamentally transformed the treatment landscape for a broad range of malignancies (1–5). Despite substantial improvements in survival outcomes, responses to ICIs remain highly heterogeneous, reflecting the complex interplay between tumor-intrinsic factors, host immune status, and external immune-modulating influences (6). Understanding factors that may modify the efficacy of ICIs is therefore of significant clinical relevance.

Since late 2020, mass vaccination against coronavirus disease 2019 (COVID-19) has been rapidly implemented worldwide, including among patients with cancer receiving systemic anticancer therapies (7). COVID-19 vaccines, particularly mRNA- and adenoviral vector–based platforms, are designed to induce robust innate and adaptive immune responses through antigen presentation, T-cell priming, and cytokine activation (8, 9). From an immunological perspective, these immune-stimulating effects raise an important clinical question: whether COVID-19 vaccination may influence the antitumor activity of immune checkpoint blockade. While vaccination could theoretically enhance ICI efficacy by promoting immune activation, alternative hypotheses suggest that immune competition, transient immune exhaustion, or altered inflammatory states could attenuate treatment benefit. As a result, the interaction between COVID-19 vaccination and ICI efficacy remains biologically plausible but clinically uncertain.

To date, accumulating evidence has primarily focused on the safety of COVID-19 vaccination in patients receiving ICIs. Several studies have consistently demonstrated that COVID-19 vaccination is not associated with an increased risk of immune-related adverse events in this population, providing reassurance regarding vaccine safety (10–12). In contrast, whether COVID-19 vaccination influences the therapeutic efficacy of ICIs has not been systematically evaluated. Specifically, no meta-analysis has comprehensively assessed the association between COVID-19 vaccination and key oncologic outcomes in patients treated with ICIs.

Multiple observational studies and real-world cohorts have investigated the relationship between COVID-19 vaccination and clinical outcomes in patients receiving ICIs, yet the findings remain inconsistent (13–15). Some studies reported comparable response rates and survival outcomes between vaccinated and unvaccinated patients, whereas others suggested potential differences in progression-free or overall survival. These discrepancies may be attributed to limited sample sizes, heterogeneity in tumor types, variability in vaccination timing relative to ICI initiation, differences in vaccine platforms, and residual confounding inherent to observational designs. Importantly, no randomized controlled trials were specifically designed to address this question, and individual studies are often underpowered to draw definitive conclusions.

Given the widespread use of COVID-19 vaccines and expanding indications for ICIs across multiple malignancies, a rigorous synthesis of available evidence regarding oncologic efficacy is urgently needed. We therefore conducted this systematic review and meta-analysis to evaluate the association between COVID-19 vaccination and ICI efficacy in patients with cancer, aiming to provide evidence-based insights for clinical decision-making in the era of routine vaccination.

## Methods

### Study design and reporting standards

This meta-analysis was conducted in accordance with the Preferred Reporting Items for Systematic Reviews and Meta-Analyses (PRISMA) 2020 statement (16). The study protocol was prospectively developed, including predefined eligibility criteria, outcomes of interest, and statistical methods, and was registered in the International Prospective Register of Systematic Reviews (PROSPERO; registration number: CRD420261277938).

### Literature search strategy

A comprehensive literature search was performed in PubMed, Embase, and Scopus from January 2020 to December 31, 2025. The search strategy combined controlled vocabulary terms (Medical Subject Headings [MeSH] and Emtree terms) and free-text keywords related to COVID-19 vaccination, immune checkpoint inhibitors, and malignancy. No restrictions were applied with respect to tumor type or geographic region. The full search strategies for each database are provided in the **Supplementary Table 1**.

In addition, the reference lists of relevant reviews and eligible articles were manually screened to identify any additional studies that may have been missed by the electronic search.

### Eligibility criteria

Studies were eligible for inclusion if they met all of the following criteria:

Population: Adult patients (≥18 years) with a histologically or cytologically confirmed malignancy treated with ICIs, including PD-1, PD-L1, and/or CTLA-4 inhibitors.

Exposure: Receipt of at least one dose of a COVID-19 vaccine, regardless of vaccine platform.

Comparator: Patients receiving ICIs who were not vaccinated against COVID-19.

Outcomes: Reported at least one oncologic efficacy outcome, including progression-free survival (PFS), overall survival (OS), objective response rate (ORR), or disease control rate (DCR).

Study design: Observational studies, including prospective or retrospective cohort studies and real-world analyses.

Studies were excluded if they were reviews, editorials, case reports, or conference abstracts without sufficient data, or if they focused exclusively on vaccine safety without reporting oncologic efficacy outcomes. In addition, studies that compared different COVID-19 vaccination statuses (e.g., fully vaccinated *versus* partially vaccinated) without including an unvaccinated comparator group were excluded. When multiple publications reported overlapping patient cohorts, the most comprehensive or most recent study was included.

### Data extraction

Two investigators independently screened titles and abstracts for eligibility, followed by full-text review of potentially relevant studies. Data were independently extracted using a standardized data collection form, with discrepancies resolved by consensus.

Extracted data included: first author, year of publication, study design, country, tumor type, sample size, type of ICI, COVID-19 vaccine platform, timing of vaccination relative to ICI initiation, and reported efficacy outcomes (ORR, DCR, PFS, OS), along with corresponding effect estimates and confidence intervals when available.

### Definition of outcomes and exposure

The primary outcomes of interest were PFS and OS. Secondary outcomes included ORR and DCR, as defined by each individual study according to standard response criteria.

COVID-19 vaccination status was defined based on study-specific criteria and typically reflected receipt of at least one vaccine dose either before or during ICI treatment. Given heterogeneity in vaccination timing across studies, patients were classified according to vaccination status as reported in the original publications.

### Risk of bias assessment

The methodological quality of included observational studies was assessed independently by two investigators using the Newcastle–Ottawa Scale (NOS) (17). Studies were evaluated across three domains: selection of study groups, comparability of cohorts, and ascertainment of outcomes. Studies with a NOS score of ≥7 were considered to be of high methodological quality. Any disagreements in quality assessment were resolved through discussion.

### Statistical analysis

For time-to-event outcomes (PFS and OS), hazard ratios (HRs) with corresponding 95% confidence intervals (CIs) were extracted directly from the original studies. For studies in which 95% CIs were not explicitly reported, confidence intervals were estimated based on the reported *P* values using established statistical methods, as previously described (18). For dichotomous outcomes (ORR and DCR), odds ratios (ORs) with 95% CIs were calculated.

Pooled effect estimates were calculated using a random-effects model (DerSimonian–Laird method), accounting for between-study heterogeneity. Statistical heterogeneity was assessed using the Cochran *Q* test and quantified with the *I²* statistic, with *I²* values of >50% indicating substantial heterogeneity.

Prespecified subgroup analyses were conducted, when feasible, based on vaccine platform and tumor type. Sensitivity analyses were performed by sequentially excluding individual studies to assess the robustness of the pooled estimates. Publication bias was evaluated using funnel plots and Egger’s regression test.

All statistical analyses were performed using R software (version 4.3.2), and a two-sided *P* value of <0.05 was considered statistically significant.

## Results

### Literature search and study characteristics

The systematic literature search yielded a total of 1,378 records across PubMed, Embase, and Scopus. After removal of duplicates and screening of titles and abstracts, 12 potentially eligible articles underwent full-text review. Ultimately, 10 observational studies met the predefined inclusion criteria and were included in the final meta-analysis (12–15, 19–24). The PRISMA flow diagram illustrating the process of identifying eligible studies is presented in **Supplementary Figure 1**.

The included studies were published between 2022 and 2025 and comprised 4,929 patients receiving ICIs therapy, of whom approximately half had received at least one dose of a COVID-19 vaccine. Study designs included nine retrospective cohort studies and one prospective cohort study. The studies were conducted across multiple geographic regions, including China, Japan, the United States, and Europe. Tumor types included non–small cell lung cancer (NSCLC), melanoma, nasopharyngeal carcinoma (NPC), head and neck squamous cell carcinoma (HNSCC), and other solid tumors. COVID-19 vaccines administered across studies included mRNA-based vaccines, inactivated vaccines, and mixed vaccine platforms. Vaccination timing relative to ICIs initiation varied substantially among studies. Detailed characteristics of the included studies are summarized in **Table 1**.

**Table 1:** Characteristics of the included studies.

Studies	Study design	Country	Sample sizes (vacc *vs* unvacc)	Cancer type	Cancer stage	ICI used (Line of therapy)	Vaccine platform	Number of doses, booster administration	Time window of vaccination	Outcomes reported (vacc *vs* unvacc)
Mei et al., 2022 (13)	Retrospective cohort study	China	1060 (530/530)	Multiple solid tumors	Metastatic (99.2%)	Camrelizumab (Mixed)	Inactivated vaccine	At least one dose. Booster information not reported.	Median 42.3 days (IQR 6.1–81.5) post-ICI initiation	ORR: 25.3% *vs* 28.9% DCR: 64.5% *vs* 67.0%
Hayashi et al., 2023 (19)	Retrospective cohort study	Japan	90 (54/36)	NPC	Recurrent or metastatic	Nivolumab (NR)	mRNA vaccine	At least one dose. Booster information not reported.	7 days post-ICI initiation	ORR: 11.1% *vs* 11.1% DCR: 64.5% *vs* 67.0%
Hua et al., 2023 (20)	Retrospective cohort study	China	1119 (746/373)	NPC	Recurrent metastatic	PD-1 inhibitors (First-line)	Inactivated vaccine	At least one dose. Booster information not reported.	Pre-/post-ICI initiation: median 105.0 days (range: -24 to 154)	ORR: 59.0% *vs* 35.7% DCR: 80.2% *vs* 72.5%
Khaddour et al., 2023 (21)	Retrospective cohort study	United States	151 (94/57)	NSCLC and HNSCC	Metastatic	NR	mRNA vaccine	At least one dose. Booster information not reported.	NR	PFS: 0.58 (0.34–1.00) OS: 0.49 (0.24–0.99)
Qian et al., 2023 (22)	Retrospective cohort study	China	104 (25/79)	NSCLC	Stage III–IV	PD-(L)1 inhibitors (Mixed)	Inactivated vaccines	At least one dose. Booster information not reported.	NR	ORR: 28.0% *vs* 11.4% DCR: 88.0% *vs* 54.4% PFS: 0.26 (0.09–0.76) OS: 0.19 (0.04–0.80)
Li et al., 2024 (23)	Retrospective cohort study	China	256 (65/131)	Multiple solid tumors	Stage IV	PD-(L)1 inhibitors (NR)	Inactivated vaccine	At least one dose. Booster information not reported.	NR	ORR: 4.6% *vs* 0.8% DCR: 66.2% *vs* 33.1% PFS: 0.54 (0.36–0.82) OS: 0.45 (0.24–0.86)
Fabbri et al., 2025 (15)	Prospective cohort study	Italy	204 (102/102)	NSCLC	Metastatic (Stage IV)	Pembrolizumab, cemiplimab (First-line)	mRNA vaccine	All patients received a booster dose (third dose).	From 60 days pre- to 30 days post-ICI initiation	PFS: 0.88 (0.66–1.18) OS: 0.81 (0.59–1.09)
Grippin et al., 2025 (14)	Retrospective cohort study	United States	884 (180/704)	NSCLC	Stage III/IV	Pembrolizumab (First-line)	mRNA vaccine	At least one dose. Booster information not reported.	Within 100 days of ICI initiation	OS: 0.51 (0.37–0.71)
			210 (43/167)	Melanoma	Metastatic	PD-(L)1 inhibitors (Mixed)				PFS: 0.63 (0.40–0.98) OS: 0.37 (0.18–0.74)
Luo et al., 2025 (12)	Retrospective cohort study	China	394 (150/244)	Lung cancer	Stage I–IV (majority advanced)	PD-(L)1 inhibitors (Mixed)	Mixed (84.67% inactivated, 15.33% mRNA)	At least one dose. Booster information not reported.	NR	PFS: 0.89 (0.61–1.29)
Wang et al., 2025 (24)	Retrospective cohort study	United States	241 (NR/NR)	NSCLC	Stage IV	NR	NR	At least one dose. Booster information not reported.	Within 100 days of ICI initiation	OS: 0.43 (0.28–0.66)
			216 (NR/NR)	Melanoma	Stage IV	NR	NR			OS: 0.53 (0.31–0.90)

ORR, Objective Response Rate; DCR, Disease Control Rate; PFS, Progression-Free Survival; OS, Overall Survival; ICI, Immune Checkpoint Inhibitor; PD-1, Programmed Cell Death Protein 1; PD-L1, Programmed Death Ligand 1; NR, Not Reported; IQR, Interquartile Range; NPC, Nasopharyngeal Carcinoma; NSCLC, Non-Small Cell Lung Cancer; HNSCC, Head and Neck Squamous Cell Carcinoma; vs, versus; post-, after; pre-, before; mRNA, Messenger Ribonucleic Acid.

### Risk of bias assessment

The methodological quality of included studies was assessed using the NOS. Overall, study quality was moderate to high, with NOS scores ranging from 4 to 9 (**Supplementary Table 2**). Five studies were considered high quality (NOS ≥7), while the remaining five studies were of moderate quality, primarily due to limitations related to cohort comparability and follow-up duration.

### COVID-19 vaccination and PFS

**Figure 1:**
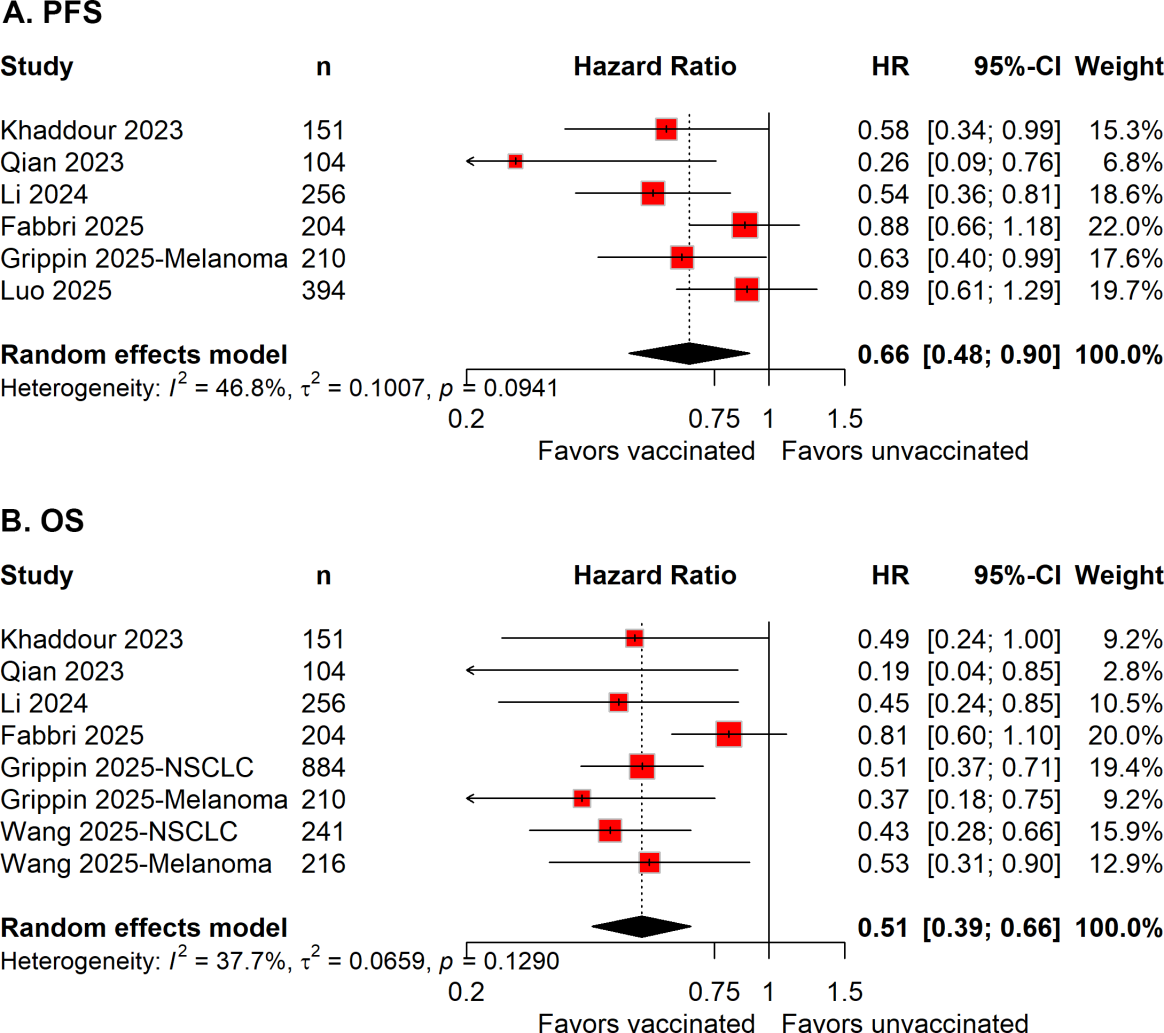
Forest plot of the meta-analysis evaluating the association between COVID-19 vaccination and **(A)** progression-free survival (PFS) and **(B)** overall survival (OS) in cancer patients treated with immune checkpoint inhibitors.

Six studies (*n* = 1,319) reported PFS outcomes (12, 14, 15, 21–23). Meta-analysis showed that COVID-19 vaccination was associated with a significant improvement in PFS (pooled HR = 0.66, 95% CI 0.48–0.90; **Figure 1A**). Heterogeneity among studies was moderate (*I^2^* = 46.8%), reflecting differences in tumor types, vaccine platforms, and study designs.

### COVID-19 vaccination and OS

Six studies with eight cohorts (*n* = 2,266) reported data on OS (14, 15, 21–24). Pooled analysis using a random-effects model demonstrated that COVID-19 vaccination was associated with significantly improved OS compared with no vaccination (pooled HR = 0.51, 95% CI 0.39–0.66; **Figure 1B**). Moderate heterogeneity was observed across studies (*I^2^* = 37.7%).

### ORR and DCR

**Figure 2:**
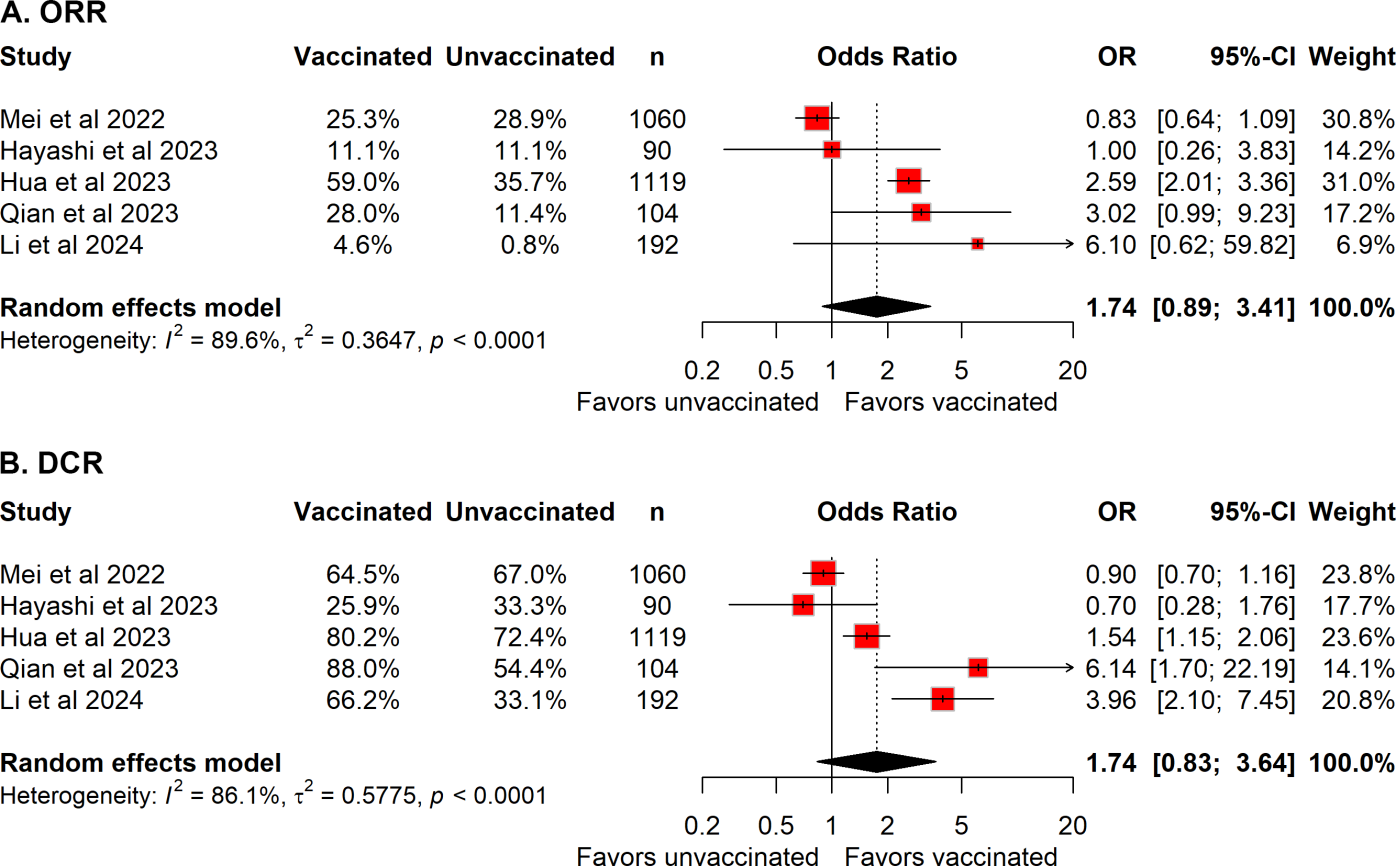
Forest plot of the meta-analysis evaluating the association between COVID-19 vaccination and **(A)** objective response rate (ORR) and **(B)** disease control rate (DCR) in patients receiving immune checkpoint inhibitors.

Five studies reported ORR and DCR data (13, 19, 20, 22, 23). Compared with unvaccinated patients, COVID-19–vaccinated patients showed a numerically higher ORR; however, this difference did not reach statistical significance (pooled OR = 1.74, 95% CI 0.89–3.41; **Figure 2A**). Similarly, pooled analysis of DCR demonstrated a trend toward improved disease control in vaccinated patients, although the association was not statistically significant (pooled OR = 1.74, 95% CI 0.83–3.46; **Figure 2B**).

### Subgroup analyses

Subgroup analyses were conducted to explore potential sources of heterogeneity.

**Figure 3:**
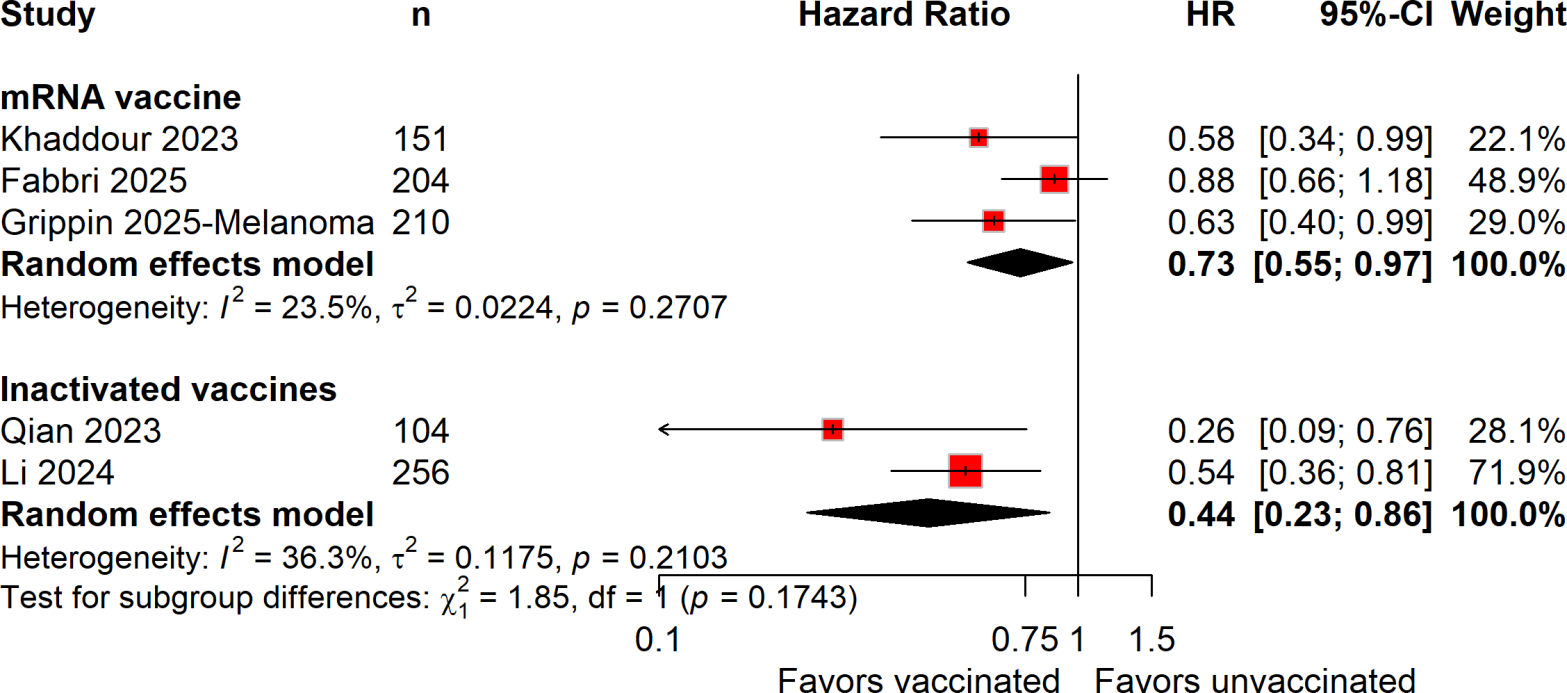
Forest plot of subgroup analysis for progression-free survival (PFS), stratified by COVID-19 vaccine platform, comparing vaccinated and unvaccinated patients treated with immune checkpoint inhibitors.

**Figure 4:**
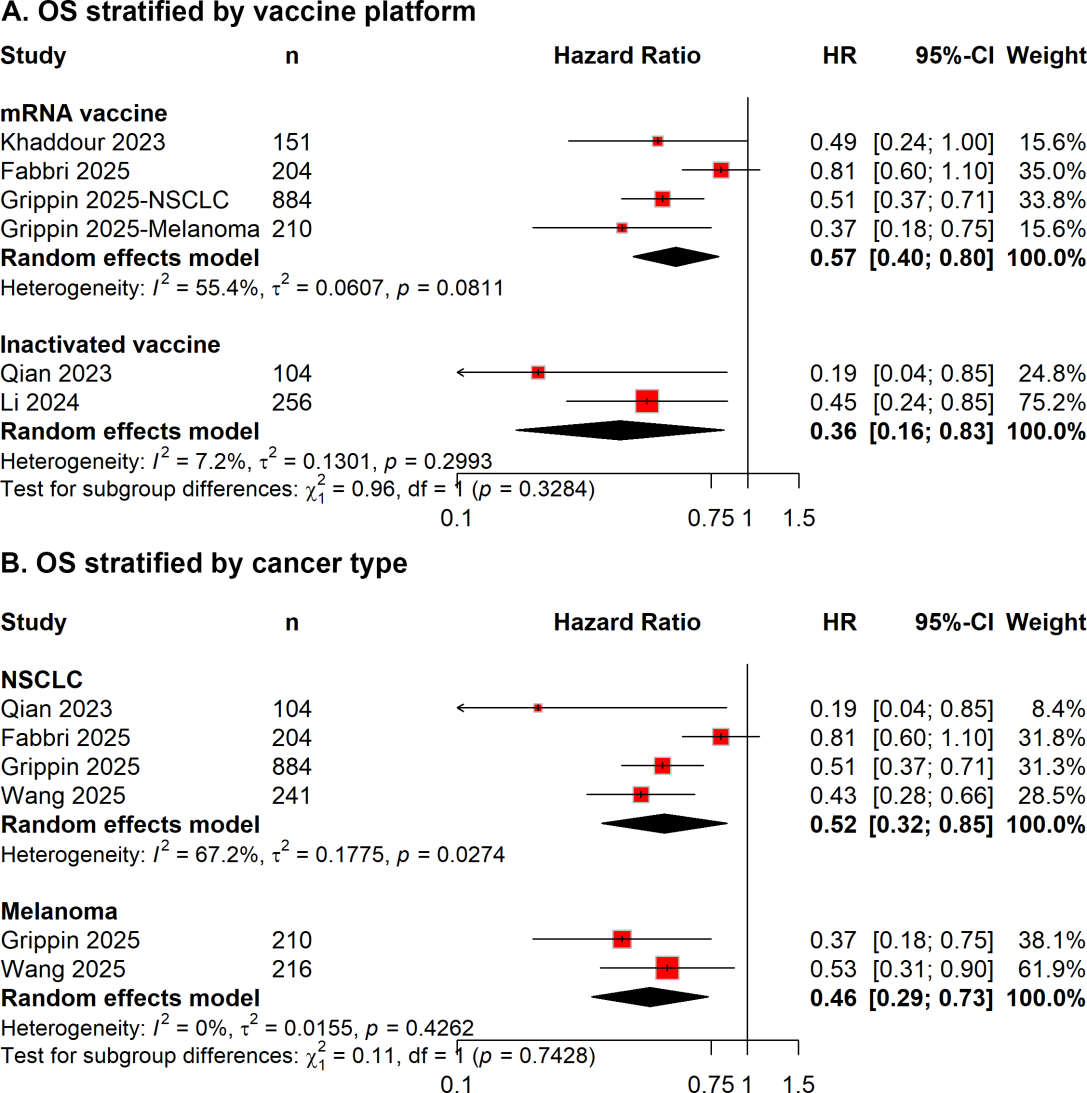
Forest plot of subgroup analyses for overall survival (OS), stratified by **(A)** COVID-19 vaccine platform and **(B)** cancer type, comparing vaccinated and unvaccinated patients treated with immune checkpoint inhibitors.

In subgroup analyses stratified by COVID-19 vaccine platform, consistent associations between vaccination and improved survival outcomes were observed across vaccine platforms. Both mRNA-based and inactivated vaccines were associated with improved PFS (mRNA-based: HR = 0.73, 95% CI 0.55–0.97; inactivated: HR = 0.44, 95% CI 0.23–0.86; **Figure 3**) and OS (mRNA-based: HR = 0.57, 95% CI 0.40–0.80; inactivated: HR = 0.36, 95% CI 0.16–0.83; **Figure 4A**). Tests for interaction did not show statistically significant differences between vaccine platforms (*P* for interaction = 0.1743 for PFS and 0.3284 for OS).

In subgroup analyses stratified by tumor type, improved OS associated with COVID-19 vaccination was observed across major cancer types, including NSCLC and melanoma, although effect sizes varied (NSCLC: HR = 0.52, 95%CI 0.32–0.85; melanoma: HR = 0.46, 95% CI 0.29–0.73; **Figure 4B**). No statistically significant interaction was observed across tumor types (*P* for interaction = 0.7428).

### Sensitivity analyses

Sensitivity analyses were performed using a leave-one-out approach for both PFS and OS. Sequential exclusion of individual studies did not materially alter the pooled effect estimates, indicating that the overall results were robust and not driven by any single study (**Supplementary Figure 2**).

### Publication bias

Funnel plot inspection for OS demonstrated some asymmetry, raising the possibility of publication bias (**Supplementary Figure 3**). Egger’s regression test showed a borderline *P* value (*P* = 0.0500), suggesting the possibility of publication bias.

## Discussion

This systematic review and meta-analysis of observational studies indicates that COVID-19 vaccination is associated with improved PFS and OS in patients receiving ICIs. Although pooled estimates for ORR and DCR did not achieve statistical significance, the consistent direction of associations across multiple efficacy endpoints strengthens the rationale for further investigation into whether vaccination-induced immune modulation may be associated with antitumor immunity. Importantly, given the observational nature of the included studies, the present analysis is intended to characterize the consistency, direction, and magnitude of associations across heterogeneous real-world settings, rather than to establish causality.

A coherent immunological rationale may underlie these observed associations. COVID-19 mRNA vaccines elicit robust type I interferon (IFN-I) signaling and proinflammatory cytokine release, which enhance systemic antigen presentation and broaden T-cell repertoires (14, 25, 26). Such vaccine-induced immune activation might transiently modify the tumor microenvironment, potentially increasing immune infiltration and lowering the threshold for effective checkpoint blockade. Supporting this, preclinical and translational studies have demonstrated that mRNA vaccination can augment dendritic cell maturation, CD8^+^ T-cell priming, and upregulate PD-L1 expression (14). While these mechanistic insights derive primarily from non-randomized and preclinical evidence, they provide a plausible biological framework that aligns with the survival benefits observed in this meta-analysis and supports the hypothesis that vaccine-induced immune stimulation could potentially complement ICI therapy, rather than impede it.

Although direct comparative data are limited, it is plausible that immunomodulatory effects could differ by vaccine platform. For instance, mRNA vaccines induce potent type IFN-I and Th1-biased immunity, which theoretically might lead to more pronounced immune activation compared with other platforms (25, 27). However, in the present analysis, no statistically significant interaction by platform was observed, and these mechanistic considerations remain speculative. The timing of COVID-19 vaccination relative to ICI initiation may represent an important modifier of treatment outcomes. However, across available observational studies, vaccination timing was heterogeneous and inconsistently defined, precluding a quantitative assessment of its impact in the present meta-analysis.

These findings are consistent with current clinical guidelines, which recommend COVID−19 vaccination for patients on ICI therapy. This recommendation is supported not only by its proven role in preventing severe SARS−CoV−2 infection but also by the absence of evidence for compromised antitumor efficacy. Furthermore, our results raise the hypothesis that vaccination may represent a potential immunomodulatory factor in the context of ICI therapy, although its clinical utility as a therapeutic strategy requires prospective validation.

Interpretation of these findings must consider several important limitations inherent to observational data. A key concern is immortal time bias, whereby patients in the vaccinated group must survive long enough to receive vaccination, potentially leading to an overestimation of survival benefits. Target trial emulation (TTE) frameworks have been proposed to mitigate this bias by aligning the start of follow-up between exposure groups, and re-analyses using such approaches have reported attenuated effect sizes (28). It should also be noted that the pooled PFS analysis was based on a relatively limited number of studies and sample size, which may reduce statistical power and affect the precision and stability of the estimated effect. In addition, residual confounding and selection bias remain important concerns. Patients who receive vaccination may differ systematically from those who do not, including in terms of health-seeking behaviors, access to care, comorbidity burden, and baseline prognosis (i.e., healthy vaccinee bias). These factors may not be fully accounted for in the original studies and could influence the observed associations. Furthermore, heterogeneity in the definition of vaccination exposure across studies—including differences in timing relative to ICI initiation, number of doses, and booster administration—may introduce misclassification and complicate the interpretation of pooled estimates. Variability in study design and analytical approaches may further contribute to between-study heterogeneity and limit comparability. Additionally, survival outcomes may be influenced by competing risks related to COVID-19 infection, including infection-related morbidity and mortality, which are difficult to disentangle from direct antitumor effects in observational datasets. Information on concurrent or prior administration of other vaccines (e.g., influenza vaccination) was not consistently reported and may introduce additional confounding. Moreover, detailed stratification of ICI regimens was not uniformly available across studies, precluding more granular subgroup analyses and potentially contributing to residual heterogeneity. Finally, evidence of potential publication bias, as suggested by funnel plot asymmetry and a borderline Egger’s test, indicates that the observed effect sizes may be overestimated, particularly if smaller studies with null or negative findings are underrepresented. Given the limited number of included studies, the ability to formally adjust for publication bias remains constrained. Therefore, while the findings of this meta-analysis are consistent and biologically plausible, they should be interpreted with appropriate caution, and causal inferences cannot be established.

Future research should prioritize prospectively designed studies that employ causal inference methods (e.g., TTE), incorporate serial biomarker profiling (tissue and peripheral), and systematically evaluate the impact of vaccine platform and timing. Such studies are essential to elucidate mechanistic pathways and optimize the integration of vaccination within immuno-oncology treatment paradigms.

## Conclusion

This meta-analysis demonstrates an association between COVID-19 vaccination and improved survival outcomes in patients receiving ICIs. While the observational nature of the data warrants cautious causal interpretation, the consistency of the signal across studies, its biological plausibility, and the robustness of the findings support the clinical compatibility and potential synergy of vaccination with ICI therapy and provide a strong rationale for further prospective investigation.

## Data Availability

The original contributions presented in the study are included in the article/**Supplementary Material**. Further inquiries can be directed to the corresponding authors.
