# Supplementary material for: COVID-19 vaccination and clinical outcomes of immune checkpoint inhibitors therapy in cancer patients: a meta-analysis of real-world studies
Source: Front Immunol. 2026 Apr 16;17:1807267. doi: 10.3389/fimmu.2026.1807267 (PMC13128565; doi:10.3389/fimmu.2026.1807267)
Supplement: Supplementary file 1 [file DataSheet1.docx]

**Supplementary Material**

**COVID-19 vaccination and clinical outcomes of immune checkpoint inhibitors therapy in cancer patients: a meta-analysis of real-world studies**

**Supplementary Table 1.** Details of search strategy.

**Supplementary Table 2.** Quality assessment of included studies through the Newcastle-Ottawa Scale.

**Supplementary Figure 1.** PRISMA 2020 flow diagram of the study selection process.

**Supplementary Figure 2.** Sensitivity analyses for progression-free survival (PFS) and overall survival (OS) using a leave-one-out approach, assessing the robustness of the pooled estimates.

**Supplementary Figure 3.** Funnel plot and Egger’s test for the assessment of potential publication bias in overall survival.

**Supplementary Table 1.** Details of search strategy.

| **Database** | **Search strategy** |
| --- | --- |
| ***PubMed*** | ("COVID-19 Vaccines"[Mesh] OR "COVID-19 vaccin*"[Title/Abstract] OR "SARS-CoV-2 vaccin*"[Title/Abstract] OR "coronavirus vaccin*"[Title/Abstract] OR "SARS-CoV-2 mRNA vaccin*"[Title/Abstract] OR "COVID-19 mRNA vaccin*"[Title/Abstract] OR BNT162b2[Title/Abstract] OR mRNA-1273[Title/Abstract] OR ChAdOx1[Title/Abstract] OR Ad26.COV2.S[Title/Abstract]) AND ( "Immune Checkpoint Inhibitors"[Mesh] OR "immune checkpoint inhibitor*"[Title/Abstract] OR ICI[Title/Abstract] OR PD-1[Title/Abstract] OR PD-L1[Title/Abstract] OR CTLA-4[Title/Abstract] OR nivolumab[Title/Abstract] OR pembrolizumab[Title/Abstract] OR atezolizumab[Title/Abstract] OR durvalumab[Title/Abstract] OR avelumab[Title/Abstract] OR ipilimumab[Title/Abstract] OR cemiplimab[Title/Abstract] OR toripalimab[Title/Abstract] OR sintilimab[Title/Abstract] OR camrelizumab[Title/Abstract] OR tislelizumab[Title/Abstract] ) AND ( "Neoplasms"[Mesh] OR cancer*[Title/Abstract] OR tumor*[Title/Abstract] OR tumour*[Title/Abstract] OR malignan*[Title/Abstract] ) |
| ***Embase*** | ('covid-19 vaccine'/exp OR 'sars cov 2 vaccine'/exp OR (covid-19 vaccin* OR sars-cov-2 vaccin* OR coronavirus vaccin* OR mrna vaccin* OR bnt162b2 OR mrna-1273 OR chadox1 OR ad26.cov2.s):ti,ab,kw) AND ('immune checkpoint inhibitor'/exp OR (immune checkpoint inhibitor* OR checkpoint blockade OR ici OR pd-1 OR pd-l1 OR ctla-4 OR nivolumab OR pembrolizumab OR atezolizumab OR durvalumab OR avelumab OR ipilimumab OR cemiplimab OR toripalimab OR sintilimab OR camrelizumab OR tislelizumab OR dostarlimab OR relatlimab):ti,ab,kw ) AND ('neoplasm'/exp OR (cancer* OR tumor* OR tumour* OR malignan*):ti,ab,kw) |
| ***Scopus*** | TITLE-ABS-KEY("COVID-19 vaccine*" OR "SARS-CoV-2 vaccine*" OR "coronavirus vaccine*" OR "mRNA vaccine*" OR BNT162b2 OR mRNA-1273 OR ChAdOx1 OR "Ad26.COV2.S") AND TITLE-ABS-KEY("immune checkpoint inhibitor*" OR ICI OR "PD-1" OR "PD-L1" OR "CTLA-4" OR nivolumab OR pembrolizumab OR atezolizumab OR durvalumab OR avelumab OR ipilimumab OR cemiplimab OR toripalimab OR sintilimab OR camrelizumab OR tislelizumab) AND TITLE-ABS-KEY(cancer* OR tumor* OR tumour* OR malignan*) |

**Supplementary Table 2.** Quality assessment of included studies through the Newcastle-Ottawa Scale (NOS).

| **Studies** | **Selection** | | | | **Compatibility** | **Assessment** | | | **Total stars** | **Quality** |
| --- | --- | --- | --- | --- | --- | --- | --- | --- | --- | --- |
|  | **Representativeness of the exposed cohort** | **Selection of the non-exposed cohort** | **Ascertainment of exposure** | **Demonstration that outcome of interest was not present at the start of study** | **Comparability of cohorts on the basis of the design or analysis** | **Assessment of outcome** | **Was follow-up long enough for outcomes to occur** | **Adequacy of follow up of cohorts** |  |  |
| Mei et al 2022 | ★ | ★ | ★ | - | ★★ | ★ | ★ | - | 8★ | High |
| Hayashi et al 2023 | ★ | ★ | ★ | - | - | ★ | ★ | - | 5★ | Moderate |
| Hua et al 2021 | ★ | ★ | ★ | - | ★★ | ★ | ★ | - | 7★ | High |
| Khaddour et al 2023 | ★ | - | ★ | - | - | ★ | - | - | 4★ | Moderate |
| Qian et al 2023 | ★ | ★ | ★ | - | ★★ | ★ | ★ | - | 7★ | High |
| Li et al 2024 | ★ | ★ | ★ | - | - | ★ | ★ | - | 5★ | Moderate |
| Fabbri et al 2025 | ★ | ★ | ★ | ★ | ★★ | ★ | ★ | ★ | 9★ | High |
| Grippin et al 2025 | ★ | ★ | ★ | ★ | ★★ | ★ | ★ | - | 9★ | High |
| Luo et al 2025 | ★ | ★ | ★ | - | ★ | ★ | ★ | - | 6★ | Moderate |
| Wang et al 2025 | ★ | - | ★ | - | ★ | ★ | ★ | - | 5★ | Moderate |


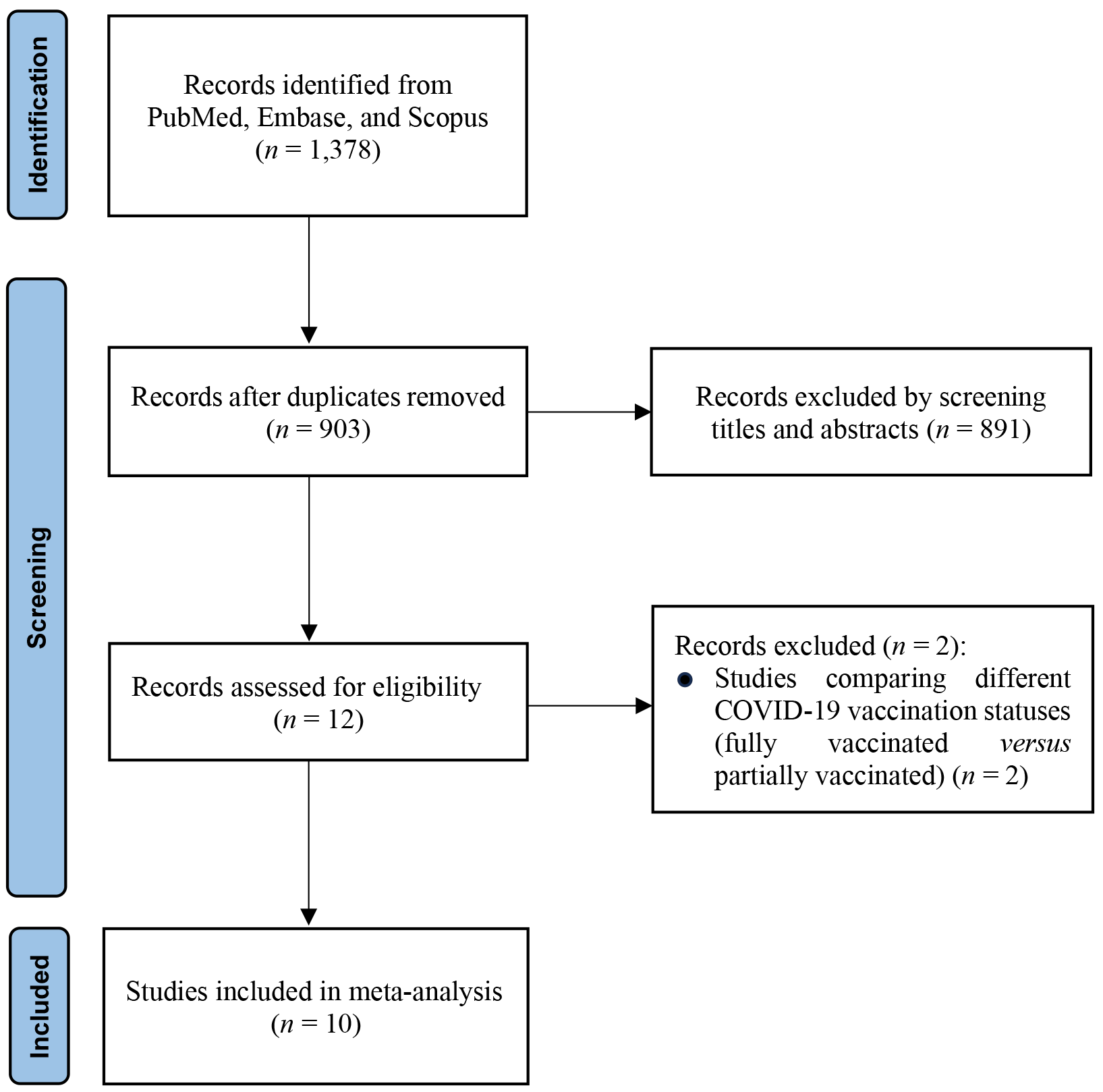


**Supplementary Figure 1.** PRISMA 2020 flow diagram of the study selection process.


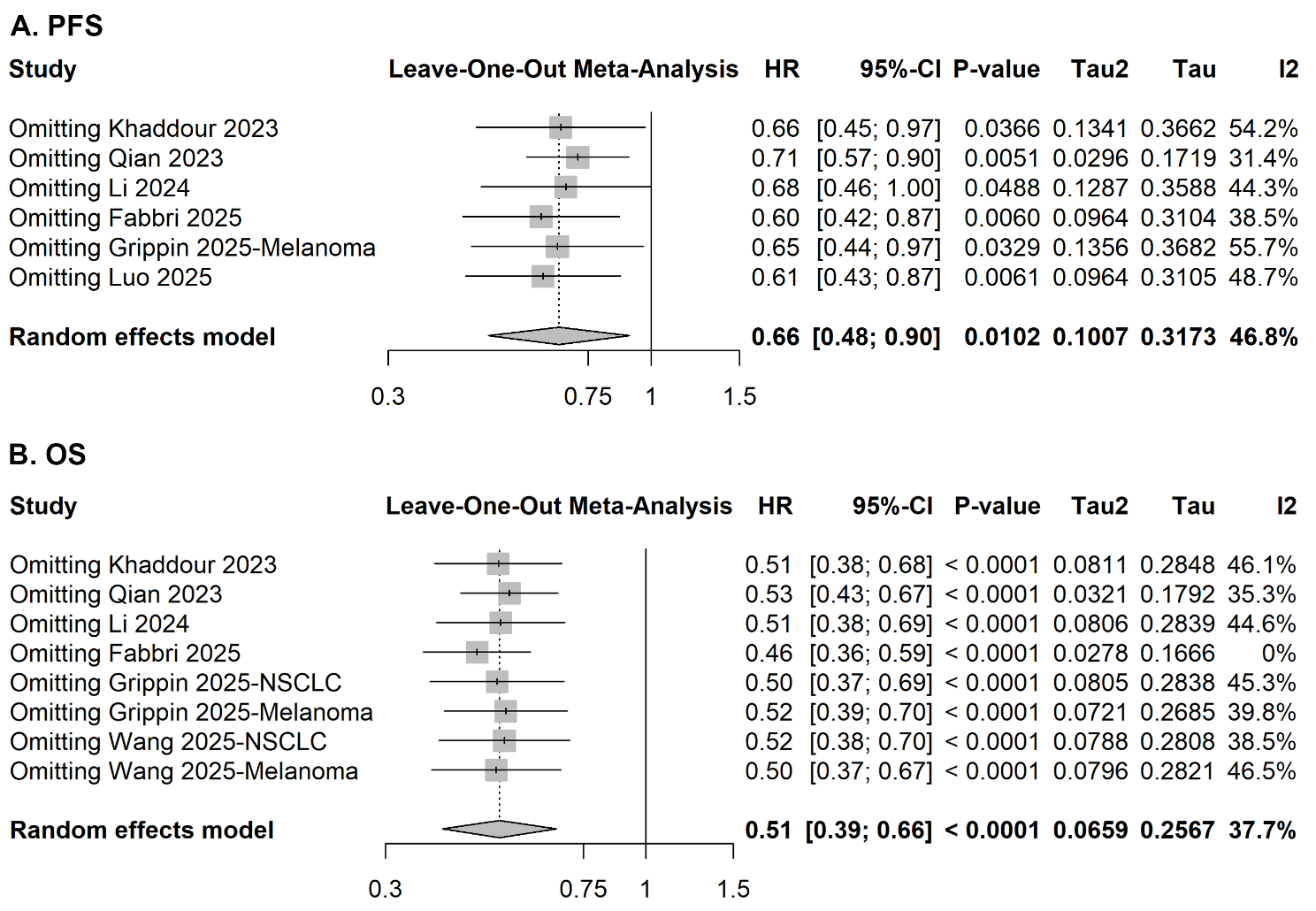


**Supplementary Figure 2.** Sensitivity analyses for progression-free survival (PFS) and overall survival (OS) using a leave-one-out approach, assessing the robustness of the pooled estimates.


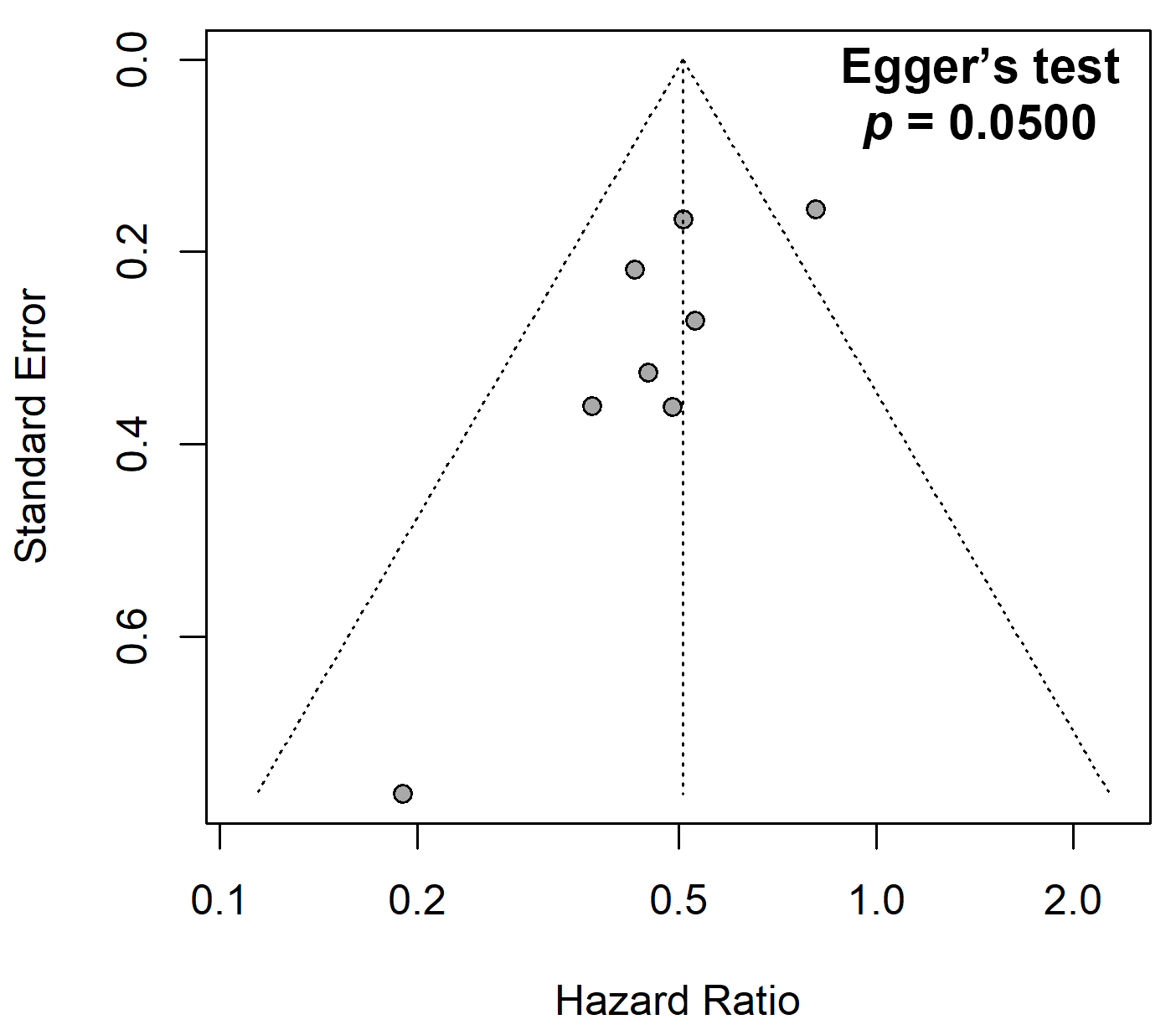


**Supplementary Figure 3.** Funnel plot and Egger’s test for the assessment of potential publication bias in overall survival.
